# Herbicide Resistance: Another Hot Agronomic Trait for Plant Genome Editing

**DOI:** 10.3390/plants10040621

**Published:** 2021-03-24

**Authors:** Amjad Hussain, Xiao Ding, Muna Alariqi, Hakim Manghwar, Fengjiao Hui, Yapei Li, Junqi Cheng, Chenglin Wu, Jinlin Cao, Shuangxia Jin

**Affiliations:** 1 Tobacco Research Institute of Hubei Province, Wuhan 430030, China; ad_hn@outlook.com (A.H.); lyp3602@163.com (Y.L.); junkey_cheng@126.com (J.C.); wuchenglin28@163.com (C.W.); 2 National Key Laboratory of Crop Genetic Improvement, Huazhong Agricultural University, Wuhan 430070, China; dxmhs1986@163.com (X.D.); munariqi@webmail.hzau.edu.cn (M.A.); 17806278731@163.com (F.H.); 3 State Key Laboratory of Conservation and Utilization of Subtropical Agro-Bioresources, South China Agricultural University, Guangzhou 510642, China; hakim_gaadwani@outlook.com

**Keywords:** base editing, prime editing, *ALS*-inhibitors, dicamba, glyphosate, weed management

## Abstract

Weeds have continually interrupted crop plants since their domestication, leading to a greater yield loss compared to diseases and pests that necessitated the practice of weed control measures. The control of weeds is crucial to ensuring the availability of sufficient food for a rapidly increasing human population. Chemical weed control (herbicides) along with integrated weed management (IWM) practices can be the most effective and reliable method of weed management programs. The application of herbicides for weed control practices calls for the urgency to develop herbicide-resistant (HR) crops. Recently, genome editing tools, especially CRISPR-Cas9, have brought innovation in genome editing technology that opens up new possibilities to provide sustainable farming in modern agricultural industry. To date, several non-genetically modified (GM) HR crops have been developed through genome editing that can present a leading role to combat weed problems along with increasing crop productivity to meet increasing food demand around the world. Here, we present the chemical method of weed control, approaches for herbicide resistance development, and possible advantages and limitations of genome editing in herbicide resistance. We also discuss how genome editing would be effective in combating intensive weed problems and what would be the impact of genome-edited HR crops in agriculture.

## 1. Introduction

The human population around the world has been expected to be increased up to 10 billion by 2050, holding tremendous pressure on current agriculture to deliver 25–70% more food production to meet the nutritional needs of the expanding population [1,2]. To meet human food requirements, global food production needs to be increased from 70 to 100% [3]. Present accumulative grain production of the world is ∼2.1 billion metric tons and an overall loss in grain yield is ∼200 million metric tons as much as 10% of this loss is due to weeds [4]. Weeds are a highly ubiquitous group of all crop pests, invading crop fields every year. In every cropping system, weeds present multidimensional problems, competing for water, space, nutrients, and sunlight that negatively affect crop production [5]. The most severe impact among all is the loss of quality and quantity of the final product [6]. Weeds not only harbor pathogens and insects that infect crop plants, but they also damage native habitats, eventually threatening native animals and plants [7]. Due to high growth capability and adaptability to multiple habitats [8], weeds can easily spread from their native environment to different regions around the globe [9], which interrupts crop development and impairs ecosystem functions [8]. Along with direct and indirect loss, they also decrease input use efficiency, cause loss of highly fertile lands, and increase cultivation costs [10]. 

Reduction in crop yield has a direct correlation with weed competition and allelopathy. Mainly, a 1-kilogram increase in growth of weeds corresponds to a decrease in 1-kilogram of crop growth [11]. Thus, weeds have been recognized as severe plant pests since the ancient times [12]. Weeds have always played a role throughout the domestication of crop plants, which led to the development of various weed control techniques [13]. At the start of weed problems in agriculture, physical and hand weeding tools were adopted for tilling the soil to manage the weeds. Later, certain other methods, such as biological and cultural approaches, were introduced. Although these approaches help in improving crop productivity and keeping weed infestations low [12], these methods have certain limitations, such as inconsistent control of weeds as well as decreased availability and increased cost of labor [14]. These methods are not always effective, lack durability, and can be expensive [12,15].

In modern agricultural systems, the management of weeds is important to ensure ample crop productivity [16], and its main purpose is to achieve maximum yield while minimizing cost. Therefore, due to the unsatisfactory weed control ability of previous methods, herbicide application became an important part of weed management programs in agriculture. Herbicide technology provides an effective and relatively cheap means of weed control, considerably reduces heavy financial burden, and contributes to increasing the average yield since the time of their adoption [17]. Currently, herbicides have been broadly applied as the primary weed control strategy for agronomic crops [18]. Based on application method, herbicides can be taken up through leaf and root absorption, which causes phytotoxic effects near the entry point, or they are translocated throughout the plant. After foliar application, the active ingredients move across several barriers, such as epicuticular waxes and leaf cuticles, until reaching the apoplast and entering in the plant cells [19]. Herbicides also enter via stomata and reach mesophyll cells [20]. The uptake in roots usually occurs in the root hairs and root tips [19]. The herbicide absorption in roots occurs in a two-step process: the initial rapid uptake through bulk water flow, and herbicide diffusion along a concentration gradient, which is a non-metabolic process. The second step is associated with metabolic process, which sees a slower entry and accumulation.

Herbicide translocation can be defined as the transport of the herbicide from the point of source to a distant target site, which involves the xylem and phloem vascular transport systems. Through translocation, the herbicide reaches both the treated and non-treated parts of the plant [19]. After entering the symplast, systemic herbicides are translocated from source leaves to younger sink leaves by the phloem [21]. The metabolisation of herbicides can occur by a natural metabolic mechanism of plant detoxification, which includes four phases [22]: (a) conversion, where the active ingredients undergo chemical modification through reduction, oxidation, oxygenation, and hydrolysis. After introducing functional groups (COOH, OH, NH_2_) the molecules become more hydrophilic and less phytotoxic [22]. The enzyme Cytochrome P450 monooxygenases (P450s) are involved in this process [23]. (b) Conjugation, where herbicide molecules or metabolites obtained from conversion are conjugated with amino acids, sugars, or the tripeptide Glutathione which increases their solubility in water and reduces phytotoxicity. One of the most important mechanisms of conjugation found in most plants includes the conjugation with glutathione (*tripeptide γ-Glu-Cys-Gly* or *GSH*), with homo-glutathione (*tripeptide γ-Glu-Cys-β-Ala* or *hGSH*), or with glucose [24]. (c) Secondary conversion and transport into vacuole. Secondary conjugation occurs in this phase and results in non-phytotoxic compounds. Further, the metabolites derived from conjugation are transported into the vacuole mainly through ABC transporters [22,24]. (d) Compartmentalization, where the metabolites from detoxification process are compartmentalized in the vacuole that may be related to the cell wall components (such as polysaccharides, lignin, pectin, and protein fractions) that form insoluble residues [24,25].

However, herbicides not only damage weeds but also affect crop plants [26]. Thus, the development of herbicide resistance (HR) in crop varieties along with the improvement of other important agronomic traits are needed, which can enhance crop production and can be helpful for farmers to manage weeds. According to the Weed Science Society of America (WSSA), herbicide resistance is defined as “the inherited ability of a plant to survive and reproduce after exposure to a dose of herbicide normally lethal to the wild type. In a plant, resistance may be naturally occurring or induced by techniques such as genetic engineering or selection of variants produced by tissue culture or mutagenesis.” Herbicide tolerance is defined as “the inherent ability of a plant to survive and reproduce after herbicide treatment. This implies that there was no selection or genetic manipulation to make the plant tolerant, it is naturally tolerant.” Sometimes HR crops are also termed as herbicide tolerant crops. Here, herbicide tolerant crops are referred to as herbicide resistant crops [27].

Conventional breeding techniques, such as mutation breeding and hybridization, have played an important role in developing HR and increasing crop productivity. The first commercial herbicide-tolerant crop obtained through conventional breeding dates back to 1984 [28]. However, whether natural variation or artificial mutation is employed, mutants are randomly generated in conventional breeding and therefore are more difficult to isolate and purify [29]. In addition, the gradual declination in natural genetic diversity in plants hugely affected crop production [30]. Thus, with the advances in next-generation sequencing technology, more and more sequenced crop genomes and newly identified gene functions lead researchers to begin transgenic breeding [29]. Although transgenic breeding remained successful, the product of this technology is GM that, unfortunately, has safety and regulatory concerns, and has not seen global acceptance [31,32]. Therefore, non-GM plants containing HR traits along with low-risk herbicides were increasingly needed by millions of multi-cropping farmers in their battle against weeds [26]. The extensive efforts of scientists led to the development of effective and presently most-appropriate genome editing tools, which have been widely used for the improvement of different traits in crop plants. 

Genome editing techniques have been effectively applied in diverse crop species to target genes for improving the average crop yield to meet the increasing demands of existing global food famine. They can provide an ecofriendly and feasible agricultural scheme to improve the varieties for better quality, higher yield, with disease resistance and HR [33,34]. This technology has revolutionized the field of crop breeding since the last few years due to its high efficacy, flexibility, simplicity, and consistency [35]. All modern genome editing tools, such as transcription activator-like effector nucleases (TALENS), zinc finger nucleases (ZFNS), clustered regulatory interspaced short palindromic repeats (CRISPR) and CRISPR-associated (Cas) techniques have been used for HR development in plants [36,37,38]. Among all gene editing techniques, CRISPR-Cas9 systems are the most effective and widely deployed to induce trait improvement in crop plants including HR [39,40]. The latest advances in genome editing have led to unique CRISPR-Cas9 tools, such as base editing­, that is more precise, efficient, and a promising tool which enables targeted point mutations via nucleotide substitution in a programmable way [41]. The CRISPR-Cas systems, particularly the base editing, have potential to generate non-GM HR crops. Additionally, non-GM plants developed through CRISPR-Cas systems have been exempted from GMO regulation in many countries [31]. Therefore, the development of non-GM HR plants by genome editing is currently the most suitable alternative to transgenic and conventional approaches, which can offer a cost-effective option to facilitate growers to manage weeds. In this review, we present the chemical method of weed control and the mechanism and approaches of HR development in plants, along with advantages and limitations of genome editing in HR development. In addition, we discuss how genome editing would be effective in combating intensive weed problems and what would be the impact of genome-edited HR crops in agriculture.

## 2. Chemical/Herbicides Method of Weed Control 

Chemical weed control is currently the most efficient, reliable, and extensively employed method of weed management in modern agricultural systems [16,42]. The first synthetic herbicide was developed in the early 1940s, which prompted a paradigm shift in agricultural weed management programs due to its selectivity and effectiveness. Presently, herbicides account for approximately 60% of pesticides used globally and most of the crop systems rely heavily on synthetic herbicides to control weeds [43]. Herbicides are small molecules (mostly <500 MW) that target specific processes in plants. The recent herbicide classification by Herbicide Resistance Action Committee (HRAC) has been categorized into various groups based on their mode of action: (1) the herbicides that inhibit activity of important enzymes, (2) herbicides targeting physiological and biochemical processes involved in photosynthesis, (3) herbicides which disturb the electron transport chain, (4) herbicides which prevent the synthesis of biological building blocks (such as amino acids, sugars, and fatty acids) and macromolecules, and (5) auxin mimics and auxin transport inhibitors [44].

However, herbicides have been classified into two types. (i) Selective/non-selective herbicides. Selective herbicides suppress the growth of target plants, leaving the desired crop unaffected. These herbicides act mainly based on phytohormones, and the selectivity can be due to differential absorption, translocation, and physiological differences between plant species. For example, 2,4-D, dicamba, and mecoprop [45]. Non-selective herbicides are not specific to some plants and affect all plant material they are applied to. For example, glyphosate, glufosinate, and paraquat are non-selective herbicides. (ii) Systemic/contact herbicides. Systemic herbicides are translocated extensively throughout the plant from the absorption to the site of action through vascular system. Systemic herbicides need a longer time than contact herbicides to kill weeds. Contact herbicides destroy only the part of plant tissue which is in contact with these herbicides and are not transferred throughout the plant. In perennial plants these are less effective, as they can regrow from roots, rhizomes, and tubers. To destroy the regrowth of the underground plant parts, repeated use of the contact herbicides is required. These herbicides have relatively rapid action. For example, bentazon and bromoxynil [46].

The major groups of selective herbicides broadly used worldwide involve Sulfonylurea herbicides, which include many Acetolactate synthase (ALS) also known as Acetohydroxyacid synthase (AHAS) -inhibiting herbicides. They represent a broad weed control potential, usage flexibility, and low application rates. Therefore, the development of Sulfonylurea resistance in crop plants can be an economical and practical way to cope with the damage caused by weeds [16]. Another group involves glyphosate that kills plants through inhibiting 5-enolpyruvylshikimate-3-phosphate synthase (EPSPS), an essential enzyme for aromatic amino acids synthesis and several other secondary products. EPSPS-catalyzed synthesis of aromatic amino acids and secondary metabolites in plants is inhibited by glyphosate via mitigating a transition state of 5-enolpyruvylshikimate-3-phosphate from phosphoenolpyruvate and shikimate-3-phosphate [47,48]. Weed control approach with glyphosate-resistant crops has been easy to use, effective, and more economical than the systems they have replaced. 

Moreover, *4-hydroxyphenylpyruvate dioxygenase* (*HPPD*)-inhibitor herbicides have been used since 1980 for selective weed control [49]. They bind slowly and very tightly with catalytic sites through the coordination of Fe atoms involved in catalysis. They inhibit the *HPPD*, an important enzyme that catalyzes the conversion of 4-hydroxyphenylpyruvic acid (4-HPPA) to homogentisic acid, and also affect the synthesis of α-tocopherol and plastoquinone [50]. In addition, glufosinate is a foliar-applied nonselective and a relatively fast-acting herbicide that degrades plants by inhibiting glutamine synthetase. The inhibition of glutamine synthetase by glufosinate causes photorespiration inhibition [51]. Glufosinate also induces rapid and mass production of ROS that results in lipid peroxidation of cell membranes casing rapid cell death [52]. Herbicides are often regarded as a relatively simple way of ensuring quick and economical method of weed control [53]. Notably, the correct use of herbicides can offer satisfactory weed control and cause little or no negative impact on the environment. Important factors of herbicide efficiency involve the selection of the correct herbicide for the weed population, following appropriate calibration procedures, and the use of herbicides at the correct time [54]. 

Because chemical weed control is the major method employed globally, the evolution of HR weeds [55] and certain environmental concerns are main constraints of this method [8]. However, the recent discovery of new herbicide modes of action as a result of current renewed interest in research and development programs observed in the agrichemical industry, as well as academic and governmental institutions, is a positive development. After three decades without the discovery of new herbicide MOA, recent studies have revealed natural phytotoxins owing herbicidal activity to novel MOA [43,56]. The mechanisms in the new mode of action involve *Lipid Biosynthesis*, *Plastoquinone Biosynthesis, Pyruvate Dehydrogenase Complex* (*PDHc*), and *Imadazoleglycerol Phosphate Dehydratase* (*IGPD*) [43]. The natural phytotoxins, like sorgoleone, involve more than one MOA that can virtually minimize the resistance development to the target site [57] by preventing photosynthesis in germinating seedlings and plants [58]. It is involved in the inhibition of PSII *in vitro* that results in plant growth reduction [59,60]. It also inhibits mitochondrial functions (Rasmussen et al., 1992), inhibits *HPPD* [61], and affects important plant processes, such as water and solute uptake [62].

Genome editing technologies, such as CRISPR-Cas9 systems, have been known to target more than one gene. Currently, base editing systems have been used to simultaneously edit *TaALS* and *ACCase* genes that offered resistance to multiple herbicides [26]. This technique could be beneficial to developing plants with higher resistance to multiple herbicides. Currently, bipyrazone has been reported as a newly developed candidate of *HPPD* inhibiting herbicides to control broadleaf weeds in the wheat growing fields in China. The study applied bipyrazone as a post-emergence herbicide in the greenhouse and in the field, which significantly controlled broadleaf weeds [63]. Thus, the development of herbicides with novel modes of action as well as crop plants with new and multiple MOA are direly needed at present to control devastating HR weed problems.

Nowadays, research efforts and funding have also been directed to integrated weed management (IWM), a holistic approach to controlling weeds that includes the application of complementary weed control methods, such as biological control, herbicide application, grazing, and land fallowing [64]. IWM has the potential to reduce weed populations to manageable level. It helps in decreasing selection pressure for weed resistance to herbicides, reducing environmental effects of individual weed control methods, and increasing sustainability for cropping systems [65]. Though a number of HR crops have been developed by genome editing, the agricultural trials with these crops are very scarce. However, HR technology can be one of the important components of IWM. The use of HR crops in IWM can provide a long-term, eco-friendly and profitable weed control system.

## 3. Mechanism and Approaches for Development of Herbicide Resistant Plants/Crops

Over the last two decades, the most suitable option for farmers to control weeds has been the use of HR crops. They became available when weed management was becoming expensive and time consuming for modern agriculture, in addition to increasing farm sizes and a decreasing number of farm workers [66]. Thus, the ability to manipulate biotechnology to generate HR crops was a big scientific innovation that led to a revolution in weed management, which offered an alternative to non-chemical weed control methods [67,68]. HR crops have been developed by several mechanisms, such as by creating modification at target sites of the herbicide to make it unable to bind on its target, by introducing or increasing herbicide inactivating or degrading enzymes into the plants, and by altering the plant to induce a mechanism that prevents the herbicide reaching a molecular target site (increasing sequestration or decreasing translocation or uptake). Metabolic inactivation or degradation is mainly the primary method of natural crop resistance against selective herbicides [69]. HR crops have laid an intense impact on the management of weeds, such as glyphosate resistant cotton (*Gossypium hirsutum*), maize (*Zea mays*), canola (*Brassica napus*), and soybean (*Glycine max*). Due to contributing to significant increases in yield and economic savings together with its efficacy and simplicity in weed management, HR technology has rapidly achieved widespread adoption [18]. However, various techniques (approaches), including genome editing, have been employed for the development of HR in plants. Despite their effectiveness, HR crops also have some environmental impacts, such as affecting agricultural practices, agronomy, management of weeds, and loss of biodiversity [70]. In the present situation of weeds, the use of herbicides seems unavoidable. However, weed control using any single approach, for example, herbicides, is not possible. The use of gene-edited HR crops specially containing new and multiple MOA in combination with IWM could be more effective to control weeds and reduce environmental impacts.

### 3.1. Mutagenesis/Mutation Breeding 

Mutation breeding is a method in which certain heritable variations provoke in the genetic material of an organism through chemical, physical (UV rays), or mobile genetic elements [71]. There are mainly three types of mutagenesis in mutation breeding: (i) induced mutagenesis, wherein mutations are induced by radiations (e.g., X-rays, gamma rays, ion beam) or chemical mutagen treatment; (ii) site-directed mutagenesis, which is the method to create specific mutations at target sites in a DNA molecule, mainly performed with PCR-based methods, traditional PCR and inverse PCR [72,73]; (iii) insertion mutagenesis, where mutations are created by DNA insertion in this process―either by genetic alteration and T-DNA insertion or activating transposable agents [74,75].

Since the 1930s, induced mutagenesis has been employed for inducing new genetic modifications [76]. Thereby, various crop plants have been developed with improved monogenic traits [77]. Many herbicide tolerant (HT) crops are produced by chemical mutagenesis and subsequent herbicide selection; for example, soybean tolerant to sulfonylurea herbicides [78], sunflower tolerant to imidazolinines and sulfonylurea [79], and wheat tolerant to sulfonylurea [80]. However, all variations utilized in commercial herbicide tolerant crops have been obtained through a single nucleotide substitution of genes, encoding proteins or enzymes targeted by herbicides. For induction of herbicide tolerance, certain HT alleles are heterozygous, some tend to be homozygous, and the rest must be stacked with another tolerance gene. All commercial HT mutations have incompletely dominant alleles, except triazine-tolerant mutation that has pleiotropic alleles, inherited maternally that are involved in many agronomic traits. HT traits can be introduced into an elite variety by crossing with a trait donor [81].

Induced mutagenesis has many shortcomings, such as the method normally being random and unstable, and beneficial mutants are unusual and mainly recessive. For selecting rare mutations, a large population size and efficient mass screening processes are needed. The dense mutation load needs comprehensive breeding for reducing background mutations and remove chimeras [76]. The possibility for simultaneous variations of more than one gene is very low [77]. In addition, transcription factors are involved to control many quantitative traits [82] and the variations in these genes can result in severe impacts on transcriptional function of their downstream targets that may explain the effects on quantitative modifications [77]. Therefore, the variations at specific sites within the plant genome instead of random non-specific variations (such as by chemical or radiation mutagenesis) had long been desired by crop producers. Consequently, the subsequent emergence of genetic engineering technology made it possible to precisely and rapidly introduce specific variations within the target gene to induce gene silencing or gene expression [83].

### 3.2. Transgenic Approach Including the Over-expression

The era of transgenic crops generated by recombinant DNA technology started in 1995. Although genetic engineering involves myriads of complex techniques, the basic principles are relatively simple. Genetically engineered, or transgenic, crops are developed through five major principles: (i) DNA extraction from organisms containing genes of interest, (ii) gene cloning, (iii) mass-construction of cloned genes in host cells, (iv) transformation (viz. *Agrobacterium tumefaciens* or gene gun), and (v) expression of introduced genes in subsequent generations. After the desired gene is stably inherited and expressed in subsequent generation, the plant is known as a transgenic plant [84]. Transgenic crops are known as genetically modified (GM) crops and the first HR GM crops commercially introduced in agriculture include bromoxynil-resistant cotton and glufosinate-resistant canola [85]. This technology brought a new revolution in weed management systems by introducing the first glyphosate-resistant crop in 1996 [18], which became the most significant transgenic crop. In modern agriculture, glyphosate-resistant crop cultivation has been the most rapidly accepted crop technology [86]. GM crops have upheld a higher adoption rate by growing on 191.7 million hectares in 2018 in 26 countries, including 18 countries regarded as biotech mega-countries producing at least 50,000 hectares. The USA was the highest producer of GM crops with 75 million hectares, followed by Brazil growing 51.3 million hectares of GM crops globally [87].

Tolerance to herbicide in glyphosate-resistant crops is from the expression of a modified form of EPSPS, which has substantially reduced binding affinity to glyphosate at the target site of the enzyme [47]. To confer resistance to glyphosate, various glyphosate-metabolizing enzymes are characterized and successfully employed in several crops to develop glyphosate tolerance; for example, increased glyphosate resistance (*igrA*), *glyphosate oxidoreductase (GOX), glycine oxidase (GO), D-amino acid oxidase (DAAO)* [88], and *glyphosate N-acetyltransferase (GAT),* which detoxifies glyphosate via N-acetylation [89]. In addition, many plants have also been modified by one of the two genes isolated from bacteria, such as bar or pat from *Streptomyces* spp. providing resistance against glufosinate-based herbicides. These genes encode *PAT (phosphinothricin acetyl transferase),* which degrades l-PPT. Other transgenes involved in HR crops confer resistance to 2,4-D (*aad-1* and *aad-12* genes), dicamba (dmo gene) or *ALS* inhibitors (*gm-hra* gene) [70].

Tobacco plants were transformed via introducing *pG2-GAT* (a plant expression vector) harboring *G2-aroA* (encoding *EPSPS*) and gat genes to induce tolerance to glyphosate. The transgenic plants co-expressing *gat* and *G2-aroA* presented higher glyphosate tolerance than plants containing gat or *G2-aroA* alone [89]. The overexpression of *G2-EPSPS* and *GAT* (glyphosate degrading gene) in soybean conferred significant glyphosate resistance along with detoxification mechanism for exclusion of accumulated glyphosate residues in the transgenic soybean plants that led to a unique strategy to develop a robust glyphosate-resistant transgenic technology [90]. In another study, transgenic soybean developed through an *Agrobacterium*-mediated soybean cotyledon node technique revealed that *G10-EPSPS* overexpression developed higher glyphosate resistance [91]. Over-expression of *proline/173/serine* (EPSPS glyphosate tolerant gene) along with *igrA*, (glyphosate detoxifying gene) caused higher glyphosate tolerance in rice. The co-expression of *OsmEPSPS* and *igrA* genes offered a dual advantage leading to detoxification and tolerance to glyphosate in transgenic rice [88]. In a recent study, Achary and colleagues introduced multiple amino acid substitution in rice *EPSPS* genes (*T173I* + *P177S*; *TIPSOsEPSPS* and *G172A* + *T173I* + *P177S*; *GATIPS-OsEPSPS*). The transformed genes containing double substitution mutation were overexpressed in rice, which developed significantly higher tolerance to glyphosate under the control of maize ubiquitin (ZmUbi) promoter. Interestingly, the transgenic rice plants presented 17 to 19% higher grain production along with greater tryptophan and phenylalanine contents in transgenic seeds compared to their wild type [92]. 

However, the products of transgenic approach are GM, and the food safety, environmental, and biosafety management procedures for GM products are cumbersome [93]. Although the safety regulation of genome editing products is still controversial [32], the edited plants without the introduction of exogenous genes [94] have not been regulated in many countries, offering an advantage over plants generated by transgenic approaches [95].

### 3.3. Genome Editing for Development of Herbicide Resistant Crops

**Figure 1:**
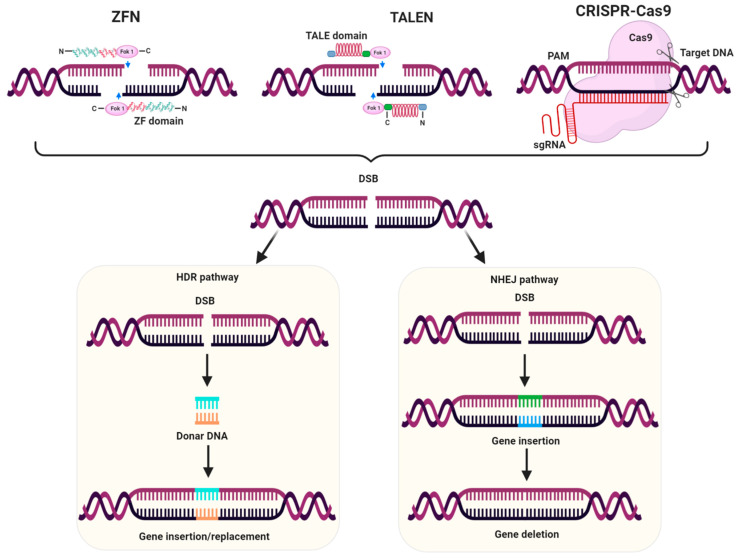
**Mechanism of genome editing tools.** These genome editing tools employ sequence-specific nucleases to recognize a particular DNA target sequences to generate double stranded breaks (DSBs). zinc finger nucleases (ZFNs) are targetable DNA cleaving proteins applied to cleave DNA sequences at any site. TALENs trigger DSBs at target site that induces DNA damage response pathways, leading to genome modification. CRISPR-Cas9 is an RNA-guided endonuclease directed by guide RNA (gRNA), and it binds at the target site adjacent to the protospacer adjacent motif (PAM) and creates a DSB. To repair the DSBs, two repair mechanisms are used by the cell: non-homologous end joining (NHEJ), which creates indels leading to a loss-of-function mutation, and homology-directed repair (HDR), which involves the introduction of a template DNA to repair DSBs that results in the correction of pre-existing mutations.

Genome editing is the collection of advanced molecular biology techniques that offer precise and efficient modifications in the targeted genomic sequences. Currently, genome editing has modernized biological research, providing new possibilities for editing the genomes of living organisms. Many genome editing techniques have been explored to edit simple and complex genomes [96,97,98]. It is significantly accelerating the progress of crop breeding, presenting a new era of genome editing-mediated plant breeding. It has enabled the introduction of desired traits in crop plants in order to improve yield, resistance to pests and herbicides, adaptation to climatic changes, and other stresses [99,100]. The major genome editing tools involve TALENs, ZFNs, and CRISPR-Cas9 systems (Figure 1), which provide simplicity and ease of targeted gene modification [98]. 

ZFNs are targetable DNA cleaving proteins used to cut DNA sequences at any site. TALENs trigger double stranded breaks (DSBs) at target site that induce DNA damage response pathways, leading to genome modification [101]. ZFNs and TALENs have been manipulated for genome modification in *Arabidopsis,* maize, rice, wheat, tobacco, tomato, and potato [102]. Although these genome editing tools have been widely utilized for genome editing, they still have certain limitations. The efficiency of ZFN is limited and it frequently introduces off-target modifications [103]. Construction of vectors for ZFNs and TALENs is time-taking and laborious [104], and has transfection inefficiency and design complexity [105]. However, the CRISPR-Cas system is the most powerful, robust, and currently the dominant gene editing tool due to its efficiency, accuracy, cost-effectiveness, and extensive application range in biological research [106].

**Figure 2:**
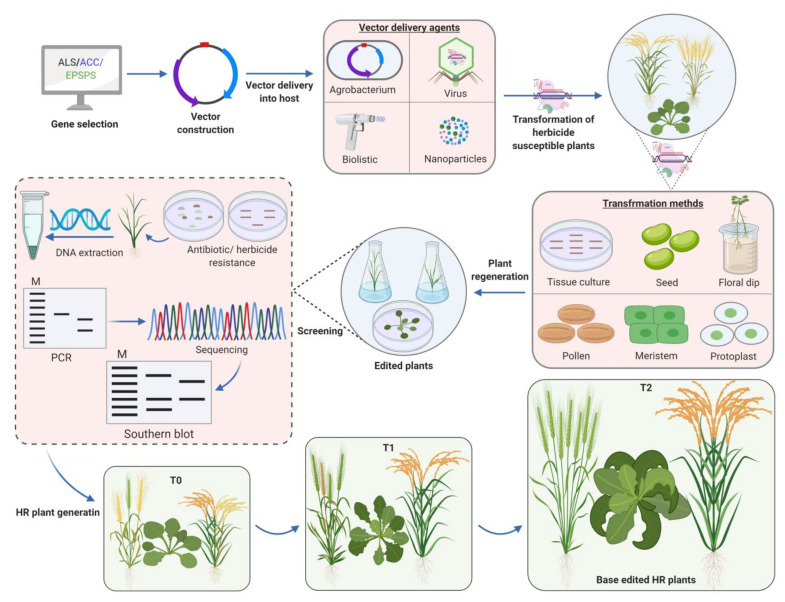
Use of genome editing for the development of herbicide resistance in plants. An herbicide resistant gene (*ALS, ACC* or *EPSPS*) is chosen and a particular target site within that gene is selected that is subsequently subjected to point mutation by base substitution. After target selection, vector construction is performed. The vector is delivered into a plant species via different methods, which is followed by plant transformation through different processes and the edited plants are regenerated. After that, the edited plants are screened for desired mutations by various methods, such as herbicide or antibiotic analysis followed by PCR, southern blotting, and sequencing. After achieving desired mutant plants (herbicide resistant plants), they are screened for particular herbicide resistance by applying the herbicide at T0, T1 or T2 generations. Typically, the base-editing generates non-transgenic (non-GM) plants.

The CRISPR-Cas system is an RNA-guided endonuclease that specifically targets DNA sequences via nucleotide base pairing. Guide RNA (gRNA) directs Cas9 to specific target site where it binds to the selected genomic locus nearby a short DNA sequence called protospacer adjacent motif (PAM), and creates a double stranded break (DSB) [31,107]. To repair the DSBs, typically two repair mechanisms have been used by a cell. Non-homologous end joining (NHEJ) mechanism, which mainly creates indels leading to a loss-of-function mutation. Another mechanism is homology-directed repair (HDR), and pre-existing mutations are corrected through this process by the introduction of a DNA sequence as a template. CRISPR-Cas9 system has proved to be a promising technology due to diverse applications in several organisms including plants. So far, this system has been employed to create targeted gene mutations, gene integration, and gene editing in numerous plant species including Arabidopsis, rice, wheat, tobacco, maize, potato, tomato, sunflower and soybean [98,108,109,110]. In addition, CRISPR-Cas9 offers revolutionary solutions to create HR plants (Table 1), (Figure 2).

The Cas9-gRNA system was effectively used for simultaneous multiple gene knockouts, endogenous gene editing, and site-specific gene integration in maize. DNA vectors expressing codon-optimized Cas9 endonuclease and sgRNAs were co-introduced with or without DNA repair templates into maize immature embryos by biolistic transformation to target different genomic loci. Modifications were subsequently identified at all sites. The editing of the ALS2 gene induced resistance to chlorsulfuron [110]. Similar system was applied in soybean to introduce targeted mutagenesis in *ALS1* to develop HR. The study targeted two genomic regions (DD20 and DD43) and obtained ~76% targeted mutagenesis and gene integration, with subsequently generated soybean plants resistant to chlorsulfuron [127]. After one year of these reports, Sun and colleagues employed CRISPR-Cas9 homologous recombination to create multiple point mutations in rice’s *ALS* gene. They simultaneously used two gRNAs along with a repair template to substitute two amino acid residues (W548L and S627I) in *ALS*. This method effectively introduced targeted mutation resulting in HR development in rice [111]. 

Many genes in plants have been targeted to achieve HR, such as *ALS, ACCase* (*acetyl-coenzyme A carboxylase*), *OsTubA2*, and *EPSPS* (Table 1). All plants have a conserved *EPSPS* motif which is important for phosphoenolpyruvate (PEP) binding or its competitive inhibitor glyphosate [118,132]. The natural substitution of two amino acids, *T102I* and *P106S* (*TIPS*), in the conserved motif was reported to develop glyphosate resistance in goosegrass [133]. Thus, glyphosate resistance in crop plants was achieved by *EPSPS* modification that became the most rapidly adopted method of HR development technology. Resistance to glyphosate has been achieved using CRISPR-Cas9 system in rice. Li [118] and colleagues introduced *TIPS* amino acid substitutions in *OsEPSPS* and achieved endogenous gene replacement and targeted gene insertion at the frequency of 2.0 % and 2.2 %, respectively. Targeted substitutions in *OsEPSPS* provided resistance to glyphosate in rice [118]. CRISPR-Cas9 in combination with ssODN was used in flax to generate herbicide tolerance by editing *EPSPS* [128].

**Figure 3:**
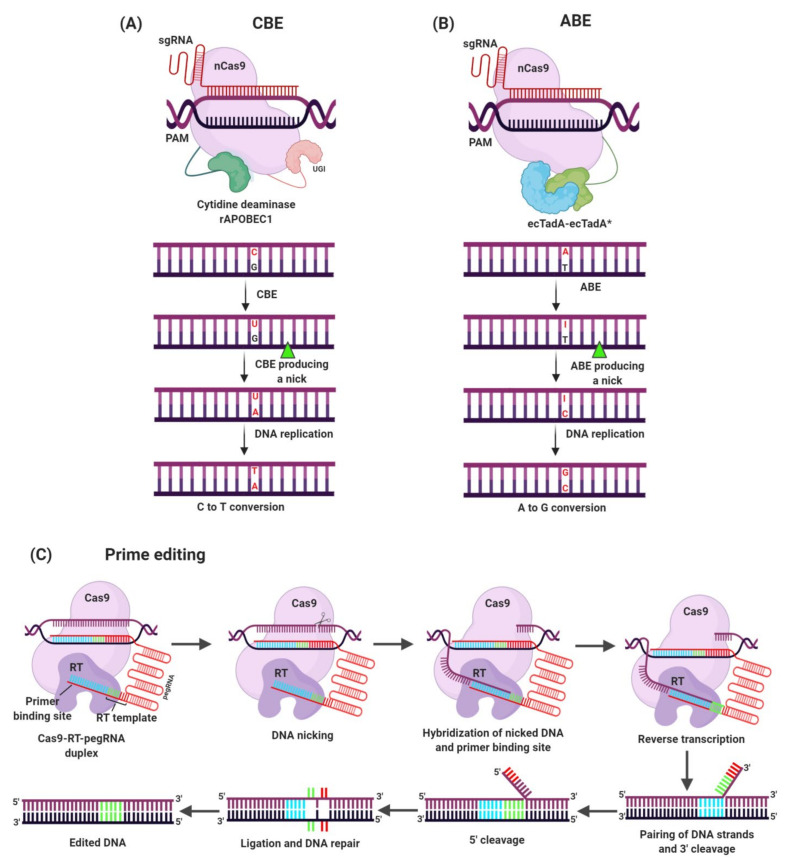
Mechanism of base editing tools. Base editing allows the introduction of precise point mutations by nucleotide substitution at specific target sites in the genome without the need of DSBs. (**A**) Cytidine deaminase base editing (CBE) is fused to an APOBEC1 cytidine deaminase that changes C-G to T-A base pair at the target loci. (**B**) Adenine deaminase base editing (ABE) is fused to an adenine deaminase that changes A-T to G-C base pairs at the target loci. (**C**) Prime editing is a newly developed base editing system enabling precise sequence substitution, insertion, and deletion. The main factor of prime editing is the fusion of nCas9 and reverse transcriptase (RT). The prime editing gRNA encodes desired edits, guiding nCas9-RT complex to the target gene sequence. The prime editor cleaves the DNA, and the cleaved DNA is hybridized to the primer binding site leading to reverse transcription. Base pairing of 3′ or 5′ flaps occur followed by DNA ligation and repair which results in DNA editing.

Although the CRISPR-Cas9 system has revolutionized the ability to introduce targeted DSBs, the HDR mechanism in plants is inefficient because of low frequency and efficacy of template DNA delivery that limits the ability to create precise mutations in the DNA sequence [134,135]. Site-specific genome editing was challenging in many eukaryotic species, involving plants [136]. Recently, base editing, a unique CRISPR-Cas9 derived system, has been developed which has enabled the introduction of precise and reproducible single base substitutions at specific target sites in the genome without the need of DSBs, donor DNA templates, HDR, and NHEJ [137]. The base editing system involves chimeric proteins consisting of a DNA targeting unit and catalytic domain that deaminate cytidine or adenine bases (Figure 3) [138]. This system has the ability to create various point mutations to screen vital amino acids and can lead to directed protein evolution in vivo [139]. Base editing platforms offer an effective and advanced method of trait improvement in crops [140] and have resolved the challenge of effectively introducing the site-specific and predictable targeted point mutations useful for crop breeding [141]. Thus, base editing has shown enormous potential for the development of HR in various crop plants (Table 1).

Single-point mutations at many conserved regions of the *ALS* gene have been identified to induce high level of resistance to herbicides in various plants [142]. The CCT codon in Pro197 of *ALS* gene located in deamination window was identified in *A. thaliana* and was targeted for base editing. The targeted DNA sequence was cloned and integrated into *Agrobacterium* GV3101 and transformed in *Arabidopsis* by floral dip method. Interestingly, the C to T conversion conferred resistance to tribenuron herbicide [124]. Cytidine base editing (CBE) was applied in watermelon to achieve C to T conversion in the *ALS* gene, and the subsequent amino acid change resulted in tribenuron HR in watermelon plants [122]. A similar system was also used in oilseed rape and the *BnALS1* gene was precisely edited at position P197 [125]. In addition, transgene free wheat was generated with multiple herbicide (imidazolinone, aryloxyphenoxy propionate and sulfonylurea) tolerances where mutations were created in *ACCase* and *ALS* genes through base editing [26]. *ACCase* is responsible for catalyzing the initial step of the biosynthesis of fatty acid. The loss-of-function modification in *ACCase* leads to severe developmental arrest in plants [115,143]. The identified *ACCase*-inhibiting herbicide resistant mutations arise in carboxyltransferase domain that directly interacts with herbicides [144]. 

Another research using the same base editing system in maize achieved the targeted amino acid substitution in two nonallelic *ZmALS1* and *ZmALS2* genes. Intriguingly, the targeted mutations in these genes conferred significantly higher resistance to chlorsulfuron herbicide in maize [95]. Another base editing tool, adenine base editing (ABE), has been used in rice by Liu and team. They integrated the rBE14/sgRNA system in rice via *Agrobacterium*-mediated transformation. The study revealed that the targeted editing of endogenous *OsTubA2* gene led to Met-268-Thr mutation that conferred resistance to dinitroaniline herbicide, i.e., pendimethalin and trifluralin. The transgene-free base-edited rice plants grown in natural light exhibited identical morphology to wild-type plants. Notably, no off-target mutations and fitness costs were observed in edited plants [114]. 

However, since the advent of base editing technology different conformational changes have been made to evolve more efficient and accurate base editing systems which could be able to introduce multiple targeted mutations within the plant genome [116,145]. Recently, such a system, the base editing-mediated gene evolution (BEMGE) was developed in rice where the combination of nCas9-based adenine and cytosine base editors and sgRNA libraries were used to generate mutations in endogenous gene [116]. The simultaneous use of multiple sgRNAs with a base editor creates robust mutations at targeted regions because of a synergistic effect that enables more progressive activity of base editors due to longer strand of ssDNA [146]. The research evolved *OsALS1* with BEMGE in rice via particle-bombardment as well as *Agrobacterium*-mediated transformation methods. Remarkably, four types of amino acid substitutions were obtained, endowing rice with varying levels of bispyribac-sodium HR [116]. Another study employed CRISPR-Cas9 and Target-activation induced cytidine deaminase (Target-AID) fusion to induce multiple herbicide-resistance point mutations through multiplexed editing to generate resistance to imazamox herbicide in rice [145]. Target-AID is a non-cleaving, nuclease deficient Cas9 complex fused to an activation-induced cytidine deaminase (AID) that produces targeted base substitution (G to A or C to T) rather than random mutagenesis [113]. 

Currently, prime editing, a novel and universal Cas9-derived precision genome editing tool, has been developed and allows precise sequence substitution, insertion, and deletion. Prime editing involves the fusion of nCas9 and RT (reverse transcriptase) along with pegRNA encoding desired edits. The prime editing gRNA guides nCas9-RT complex to the target gene sequence [147]. The prime editor cleaves the DNA, which is hybridized to the primer binding site leading to reverse transcription. Base pairing of 3′ or 5′ flaps occurs followed by DNA ligation and repair that results in DNA editing [148]. Very recently, different groups have applied prime editing to engineer HR in rice. *OsALS* was targeted to induce amino acid substitution to confer bispyribac sodium and imidazolinone resistance in rice [40,120]. Hua and colleagues used Sp-PE3 for *ALS* to introduce an S627N mutation. They found 4 out of 44 (9.1%) transgenic lines [120]. While haloxyfop resistance was developed in rice by editing *OsACC1* using the same technology [121]. 

## 4. Advantages and Disadvantages of Genome Editing in Herbicide Resistance

**Figure 4:**
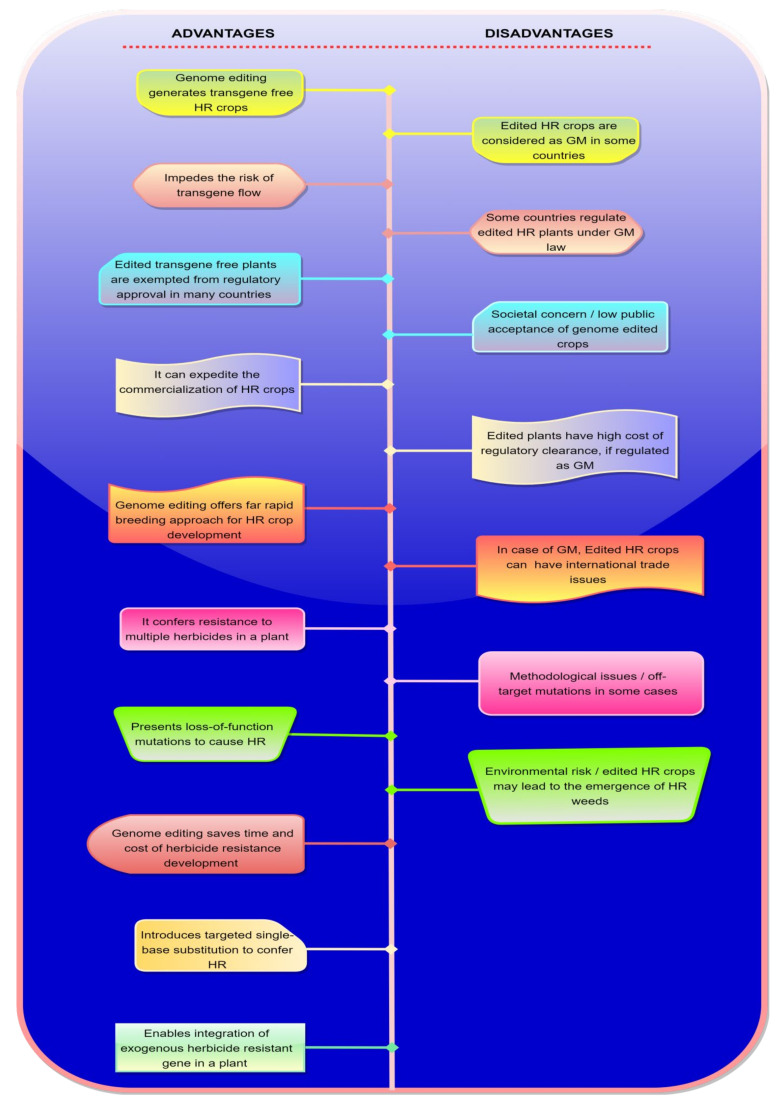
Advantages and disadvantages of genome editing in herbicide resistance. HR: Herbicide resistance, GM: Genetically modified.

Since the influence of herbicides in agriculture has raised, the call for herbicide resistant crop production is also increased. For that, the advances in genome editing, particularly the advent of CRISPR-Cas systems, has opened new avenues to accelerate HR crop development [149,150]. Genome editing offers several advantages in HR development in various ways (Figure 4). For example, genome editing enables the direct insertion of an exogenous HR gene in a plant to confer HR [88]. For example, phosphinothricin acetyltransferase (PAT) and bialaphos resistance (BAR) genes originally isolated from Streptomyces spp. have been used in plants to confer resistance to glufosinate herbicides [70]. In addition, Han and Kim have achieved the loss-of-function mutation of OXP (5-oxoprolinase) and PAI (phosphoribosylanthranilate isomerase) in plants through CRISPR-Cas9 conferring resistance to sulfamethoxazole and 6-methylanthranilate, respectively [149].

More importantly, genome editing, specially programmable base editing, presents heritable, targeted modifications leading to transgene-free HR crops [40,115,122] that are genetically non-distinguishable to that of the plants created by traditional mutagenesis [151]. Moreover, base editing offers multiple amino acids substitution of targeted genes that confers resistance to multiple herbicides [26]. Notably, unlike GM plants, the non-transgenic plants produced via CRISPR-Cas system are free from any additional regulatory approval (exempted from regulation) in several countries [152]. Therefore, genome editing can expedite the commercialization of HR crops. Compared to mutagenesis and GM approach, genome editing can save the time and cost of HR development. As the genome-edited HR crops generated without the introduction of foreign DNA do not require risk assessment, [153] this allows developers to launch new crops into the market years earlier and for millions of dollars less than GM crops [154].

Despite genome editing offering overwhelming advantages including the development of HR crops, the applications of this technology have certain limitations (Figure 4), such as the plants produced through CRISPR-Cas techniques are considered as GM in some countries that are subjected to strict regulatory procedures [35]. In the case of GM, the major factors restricting the production of HR plants include the higher costs to obtain regulatory approval along with international trade issues [155,156]. Therefore, due to these limitations, the advantage of robust technology to rapidly develop and commercialize genome-edited HR crops can be restricted.

Though genome editing has proven to be far more precise in comparison with traditional mutagenesis, the technology yet involves technical and societal concerns (public acceptance of genome-edited crops which remains the major bottleneck) along with certain environmental risks. The technical concern includes the risk of creating unintended genetic modifications in the plants owing to unprecedented integration of artificial nucleases which can lead to off-target modifications [157]. However, these technical concerns are needed to further improve the technology for its fair use in targeted trait improvements in crops. Moreover, the societal concern about the genome editing is, in part, due to a lack of knowledge of its applications and principles. The primary feature here is the difference between genome-edited, genetically modified, and transgenic plants [158,159], and the plants produced through genome editing may or may not be transgenic. Therefore, currently, the public education about genome editing principles and the knowledge of its differences from older breeding techniques is necessary for correcting and preventing the spread of suspicion and misconception [158,160]. In addition, research studies and field trials are required for the assessment of environmental and public health risks associated with gene editing crops, before launched in the market. Importantly, the cultivation of GM HR rice crop has resulted in the rise of HR weeds through hybridizing with wild species [161]. This case is highlighting the importance that the possible environmental risks of the traits introduced into the genome-edited plants must be considered prior to cultivation and commercialization of genome-edited HR crops [162].

## 5. Impact of Genome-Edited Herbicide Resistant Crops in Agriculture

Over the past 100 years, technological advances have resulted in remarkable increases in agricultural productivity [163]. The subsequent rise in molecular genetic tools has ushered in the era of genomic breeding, wherein molecular breeding and genetic engineering have gained prominence [164]. Over the past several years, both transgenic and non-transgenic HR crop development have been revolutionary approaches in agriculture; however, the transgenic HR crops remained the focus of crop improvement that presented effective and relatively novel weed management practices. Due to this reason, farmers rapidly adopted the first transgenic glyphosate-resistant crop that transformed the way many farmers managed weeds before [165,166].

Although the transgenic crops have enabled the effective weed control, they have limited public acceptance in many parts of the world that impede their widespread cultivation [163]. Moreover, the regions entirely dependant on glyphosate resistant crops led to the gradual rise of glyphosate resistant weeds [166]. To overcome these limitations, various non-transgenic HR crops have been developed by genome editing (Table 1), and few of them have been commercialized recently [167]. However, most of the studies conducted so far are on ‘proof of concept’ or improvement of the precision and delivery of the SDN [167]. Though a number of HR crops have been developed by genome editing, the agricultural trials of these crops are very scarce. 

Field experiments are conducted on genome-edited HR canola and flax. One of the first field experiments on HR canola has been done by CIBUS in the US [168]. Since the crop is non-GM, it provides alternative weed control options for GM HR crops with lower herbicide management cost [167]. Another company, Bayer Crop Science, has developed genome-edited flax with tolerance to glyphosate that was successfully marketed for around 50 million acres in 2019 [169]. Additionally, Bayer Crop Science plans to commercialize three-way herbicide tolerant XtendFlex this year. The company is also working on a novel herbicide mode of action in soybean and corn crops that would be tolerant to glufosinate and glyphosate, as well as dicamba. It can control different weed species resistant to different MOA. This novel molecule presents effective management of many herbicide-resistant weeds simultaneously. The development of a new herbicide mode of action would offer more choices to the growers for controlling weeds [169]. However, the application of genome-edited HR crops in agricultural systems, particularly with multiple HRs, can be a leading approach to combat resistant weeds thereby increasing crop yield along with soil moisture and preservation [170]. 

## 6. Concluding Remarks and Perspectives

Present agriculture is holding a dual pressure; expanding human population demands 25−70% more food production to meet the nutritional needs, and on the other hand weeds contribute to a significant yield loss in every cropping system, which is a great threat to global food security. Since weeds have evolved resistance against herbicides, weed management has become a big challenge to providing a sustainable food demand. Although many techniques have been applied in agriculture, herbicide weed management has remained the dominant technology for several years. Traditional and transgenic breeding to generate HR crops are laborious and time-consuming. Therefore, CRISPR-Cas9-mediated genome editing enables specific point mutations at target sites for gain-of-function or loss-of-function modifications—providing a great potential for targeted trait improvement in crops. Genome editing offers an alternative, the most suitable and efficient solution for the development of HR crops, which can overcome the current worsening situation of weeds in crop cultivation. The genome-edited HR crops would most probably contribute to higher crop productivity, simple and effective weed control management along with significant cost-savings.

Recent advances in genome editing have led to the emergence of novel base editing tools. Over the last three years, different base editors have been established, which have offered an obvious option for desired trait improvement in a range of crop plants (Table 1). Notably, genome editing, particularly base editing tools enable the creation of HR plants without the integration of exogenous DNA, therefore these plants are non-GM and transgene-free—similar and non-distinguishable to those developed via conventional or transgenic crop breeding [31,151]. Thus, genome-edited HR plants could have more public acceptance than GM crops. Evaluating the heritability and phenotypic stability of CRISPR-edited plants requires the elimination of CRISPR structures that is also the precondition for commercial approval of genome-edited crops. Researchers bind specifically expressed toxin proteins [171,172] and a male sterility gene [173] with the CRISPR system to eliminate the CRISPR structure in plants [174]. The technology is called TKC (Transgene Killer CRISPR). 

Importantly, genome editing has made it possible to introduce multiple-gene modifications simultaneously to induce multiple-trait improvements in a plant. For example, Zhang and colleagues have developed wheat, by genome editing, which is resistant to sulfonylurea, imidazolinone, and aryloxyphenoxy propionate herbicides [26]. In addition, Bayer Crop Science is working on soybean that would be resistant to dicamba, glufosinate, and glyphosate herbicides [169]. Thus, the multiple-HR would be another promising approach to efficiently cope with increasing HR weed problems.

Despite higher effectiveness of present genome editing HR technology, the relying of weed management on a single technique may not be enough because weeds can adapt to it within few years. Therefore, a combinatorial weed control program is necessary, such as the combination of crop rotation along with the cultivation of multiple-HR crops. However, the cultivation of genome-edited crops is restricted due to regulatory approvals in some countries, and the major challenge lies in market and commercialization—whether the consumers are willing to accept the product obtained through genome editing. This scenario requires collective effort by companies, stakeholders, universities, and the government to deliver reliable information to achieve public trust towards the non-transgenic genome-edited crops. In fact, the real benefit of this technology can only be availed if it is understood properly and accepted socially. Additionally, more research studies and field trials are required for the assessment of environmental and public health risks associated with gene editing crops, before launch in the market. Every new weed management system, such as novel gene editing HR crops, must be evaluated during development for its potential to select for resistance, and stewardship programs should be in place when the new program is introduced. The appropriate use of genome editing for agricultural purposes could provide sufficient crop production to benefit both the farmer and consumer, in addition to considerably meet human nutrition needs.

## Figures and Tables

**Table 1:** Herbicide resistant plants developed through genome editing.

Plant	Genome Editing System	Delivery Method	Target Gene	Target Herbicide	Reference
Rice	CRISPR-Cas9	Particle bombardment	*ALS*	Bispyribac- sodium	[111]
	CRISPR-Cas9	*Agrobacterium*-mediated transformation	*ALS*	Imazethapyr	[112]
	TALEN	Ballistic delivery	*OsALS*	Bispyribac- sodium	[37]
	Target-AID	*Agrobacterium*-mediated transformation	*ALS*	Imazamox	[113]
	ABE	*Agrobacterium*-mediated transformation	*OsTubA2*	Dinitroaniline	[114]
	CBE	*Agrobacterium*-mediated transformation	*OsACCase*	Gallant	[115]
	BEMGE	*Agrobacterium*-mediated and Particle bombardment	*OsALS1*	Bispyribac-sodium	[116]
	STEMEs	*Agrobacterium*-mediated transformation	*ACCase*	Haloxyfop	[117]
	CRISPR–Cas9	Particle bombardment	*EPSPS*	Glyphosate	[118]
	ABE	*Agrobacterium*-mediated transformation	*ACCase*	Haloxyfop	[119]
	Prime editing	*Agrobacterium*-mediated transformation	*OsALS*	Bispyribac sodium	[40]
	Prime editing	*Agrobacterium*-mediated transformation	*ALS*	Imidazolinone	[120]
	Prime editing	*Agrobacterium*-mediated transformation	*OsACCase1*	Haloxyfop	[121]
Watermelon	CBE	*Agrobacterium*-mediated transformation	*ClALS*	Tribenuron	[122]
Wheat	n/dCas9-PBE	Particle bombardment	*TaALS,* *ACCase*	Sulfonylurea, Imidazolinone and Aryloxyphenoxy propionate	[26]
	n/dCas9-PBE	Particle bombardment	*TaALS*	Nicosulfuron	[123]
Maize	CBE	*Agrobacterium*-mediated transformation	*ZmALS*	Sulfonylurea	[95]
	Cas9-gRNA	Particle bombardment	*ALS*	Chlorsulfuron	[110]
*Arabidopsis*	CBE	*Agrobacterium*-mediated transformation	*ALS*	Tribenuron-methyl	[124]
Oilseed rape	CBE	*Agrobacterium*-mediated transformation	*BnALS1*	Tribenuron-methyl	[125]
Tomato and Potato	CBE	*Agrobacterium*-mediated transformation	*ALS*	Chlorsulfuron	[126]
Soybean	CRISPR–Cas9	Particle bombardment	*ALS1*	Chlorsulfuron	[127]
Flax	ssODN and CRISPR/Cas9	Protoplast transfection	*EPSPS*	Glyphosate	[128]
Chile peeper	Intragenic method	*Agrobacterium*-mediated transformation	*EPSPS*	Glyphosate	[129]
Cassava	Cas9-gRNA	*Agrobacterium*-mediated transformation	*EPSPS*	Glyphosate	[130]
Potato	GVR	*Agrobacterium*-mediated transformation	*ALS1*	Imidazolinone	[131]

ABE: adenine base editing, CBE: cytidine base editing, PBE: plant base editor, BEMGE: base editing-mediated gene evolution, STEMEs: saturated targeted endogenous mutagenesis editors, GVR: geminivirus replicon.

## Data Availability

Not Applicable.
